# Application of Intelligent Ultrasound in Real-Time Monitoring of Postoperative Analgesic Nerve Block

**DOI:** 10.1155/2021/3309382

**Published:** 2021-12-09

**Authors:** Zhengwei Li, Ling Zhao, Wutao Wang, Ling Zheng

**Affiliations:** ^1^Department of Anesthesiology, The First Affiliated Hospital of Xi'an Medical University, Xi'an 710077, Shaanxi, China; ^2^Department of Anesthesiology and Perioperative Medicine, Xi'an People's Hospital (Xi'an Fourth Hospital), Xi'an 710004, Shaanxi, China

## Abstract

In order to monitor the effect of nerve block in postoperative analgesia more accurately, this paper puts forward the application research of ultrasonic real-time intelligent monitoring of nerve block in postoperative analgesia. Ultrasonic real-time intelligent monitoring of nerve block in upper limb surgery, lower limb surgery, and abdominal surgery combined with the nerve stimulator. The experiments show that there are 5 cases of adverse reactions when the nerve stimulator is only used, but no adverse reactions occur when combined with ultrasound-guided block. Continuous subclavian brachial plexus block with the ultrasound-guided nerve stimulator can clearly see the subclavian brachial plexus and its surrounding tissue structure, the direction of needle insertion in the plane, and the diffusion of narcotic drugs. The average success rate of block was up to 95.2%, which was significantly higher than that of nerve stimulator alone, and the success rate of recatheterization after the first failure was also improved. The average postoperative analgesia satisfaction was 85.6%, the average operation time was only 20 min, and the subclavian artery and pleura were avoided effectively. No pneumothorax and other complications occurred. The average success rate of ultrasound-guided subclavicular brachial plexus block in 1-2-year-old children was 97%, which was much higher than the average success rate of nerve stimulator localization with 63%. Ultrasound-guided nerve block not only directly blocks nerves under visual conditions but also helps to observe the structures around nerves and dynamically observe the diffusion of local anesthetics, which can significantly improve the accuracy and success rate of nerve block and reduce the incidence of complications.

## 1. Introduction

 Postoperative pain mainly concentrated in 24–48 h after operation, and pain itself and its influence on the normal functions of patients' breathing, circulation, digestion, and other systems were not conducive to the functional recovery of patients after operation [1]. Postoperative analgesia can alleviate and prevent the stress reaction of patients' body caused by pain. Although PCEA is effective in analgesia, it often leads to severe postoperative hypotension and urinary retention. However, postoperative anticoagulation therapy increases the risk of epidural hematoma, which affects the functional recovery [2]. Peripheral nerve block can achieve analgesia by blocking the local nerve, which is a kind of postoperative analgesia with exact effect and few complications. In recent years, it has been found that continuous peripheral nerve block is more effective than single administration for postoperative analgesia. Regional nerve block is a common method for clinical anesthesia and analgesia, and its clinical effect mainly depends on the position of the needle tip. The traditional nerve block methods are mainly the location of anatomical landmarks on the body surface, blind exploration of abnormal sensation, and nerve electrical stimulation. Because of anatomical variation, unclear body surface marks caused by obesity, or uncooperative patients, the neural location is not accurate. Even experienced anesthesiologists have a failure rate of 4%–20% [3]. Moreover, repeated detection, large dosage of local anesthetics, and strong electric shock can easily cause not only discomfort, anxiety, and panic of patients but also complications such as nerve injury, hematoma, blood vessels mistaken by local anesthetics, and pneumothorax [4]. In recent years, because of its advantages such as noninvasive and visibility, the localization technology of ultrasound imaging has created conditions for its application in nerve block, significantly improved the success rate, and reduced complications. It can not only locate accurately but also observe the local structure of the target nerve, the travel route, and direction of the puncture needle and the diffusion of the local anesthetic in real time, so as to avoid damaging the nerve tissue. There is no need for patients to express different feelings, the patients feel comfortable, and it is also suitable for infants. Ultrasound guidance is expected to become a golden technique for peripheral nerve block, which will bring convenience to clinical anesthesia and pain treatment [5]. According to the characteristics of sound waves, sound velocity = frequency × wavelength. Visible frequency is inversely proportional to wavelength. The higher the frequency, the shorter the wavelength. The smaller the minimum resolution distance (equivalent to 1/2 wavelength) is, the higher the resolution is. On the contrary, when ultrasonic waves propagate in the body, the acoustic energy will be attenuated, and its attenuation coefficient is approximately proportional to the frequency in the megahertz frequency range. The attenuation of sound energy in common human tissues from large to small is bone, scar, tendon, muscle, fat, blood, and cystic fluid [6]. The resolution of 10 MHz ultrasound can reach 0.075 nm, and the maximum penetration depth in human soft tissue is 4 cm. In recent ten years, with the development of ultrasonic examination equipment and diagnostic technology, it has been able to provide a high-frequency ultrasonic detector of 2.5–20 MHz for clinical use, and the distribution, course, and thickness of the peripheral nerve can be clearly displayed by using a high-frequency linear ultrasonic probe. In the human body, peripheral nerves are often accompanied by soft tissues such as tendons, ligaments, and blood vessels, which can be identified by color Doppler ultrasound, while tendons and ligaments are easily confused with peripheral nerves on the two-dimensional sonogram [7]. However, some scholars have found that tendons and ligaments show many thin and strong echo bands arranged in parallel on the sonogram, compared with the strong echo band of connective tissue in the nerve, the arrangement is more regular, and there is no small, round hypoechoic region formed by the nerve fiber bundle during transection. When limbs move, such as flexion or extension, the position and thickness of tendons and ligaments will change, while the size and position of nerves are relatively fixed. Sympathetic ganglion block is mainly used in the treatment of clinical painful diseases, and it is not only completely different from limb nerves in histology but also usually located in the deep part of the body and close to the great vessels, which brings great difficulties to ultrasound guidance. However, it is still possible to implement ultrasound-guided localization block according to its adjacent anatomical structure in clinics. Oral analgesia or patient controlled intravenous analgesia are the main methods used for postoperative analgesia, while oral NSaids and opioids have serious gastrointestinal side effects. The analgesic effect of PCIA is sometimes not ideal, and there are many side effects. In view of this research problem, Srebotnik-Kirbiš and LimbäCk-Stokin found that ultrasound-guided nerve block has fewer complications, and the existing reports of ultrasound-guided nerve block have no serious complications because nerves and blood vessels, pleura, and other groups can be seen on ultrasound images, as well as the diffusion of puncture needles and local anesthetics, thereby avoiding serious complications such as nerve injury, local anesthetic poisoning, and pneumothorax caused by Srebotnik-Kirbiš and LimbäCk-Stokin penetrating blood vessels and pleura. However, abnormal sensation will still occur during operation, which requires the operator to carefully and patiently insert the needle when the needle approaches the nerve, so as to reduce the occurrence of abnormal sensation. Ultrasound and nerve stimulator can also be used in combination to prevent or reduce the occurrence of abnormal sensation [8]. Finger et al. think that indications include brachial plexus and its branches (most reported), lumbar plexus and its branches (children), sciatic nerve and its branches, cervical nerve and cervical plexus, intercostal nerve, and celiac plexus, and contraindications include hematoma around the nerve or nerve position too deep, which makes neuroimaging unclear [9]. Qiu et al. used ultrasound-guided sciatic nerve and femoral nerve block for children undergoing lower limb surgery and found that the power of ultrasound-guided composition was significantly higher than that of the nerve stimulator group, and the analgesic time was significantly prolonged. Ilioinguinal/iliohypogastric nerve block (IINB) is suitable for children's inguinal and perineal operations such as indirect inguinal hernia and hydrocele [10]. Oral analgesia and patient-controlled intravenous analgesia are the main methods used for postoperative analgesia, intravenous analgesia, PCIA (patient controlled epidural Analgesia (PCEA), while oral NSAIDs and opioids have serious gastrointestinal side effects. The analgesic effect of PCIA is sometimes not ideal, and there are many side effects. On the basis of the current research, this paper proposes the application of ultrasonic real-time intelligent monitoring of nerve block in postoperative analgesia and applies ultrasonic real-time intelligent monitoring of nerve block in upper limb surgery, lower limb surgery, abdominal surgery, and other aspects combined with the nerve stimulator. The experiments showed that there were 5 cases of adverse reactions when using nerve stimulator alone, but no adverse reactions occurred when combined with ultrasound-guided block. Continuous subclavian brachial plexus block was performed with ultrasound guidance combined with the nerve stimulation instrument, and the structure of the subclavian brachial plexus nerve and its surrounding tissues, the direction of needle insertion in the plane, and the diffusion of anesthetic drugs could be clearly seen. The average success rate of block was up to 95.2%, which was significantly higher than that of nerve stimulator alone, and the success rate of recatheterization after the first failure was also improved. The average postoperative analgesia satisfaction was 85.6%, the average operation time was only 20 min, and the subclavian artery and pleura were avoided effectively. No pneumothorax and other complications occurred. The average success rate of ultrasound-guided subclavicular brachial plexus block in 1-2-year-old children was 97%, which was much higher than the average success rate of nerve stimulator localization with 63%. Ultrasound-guided nerve block not only directly blocks nerves under visual conditions but also helps to observe the structures around nerves and dynamically observe the diffusion of local anesthetics, which can significantly improve the accuracy and success rate of nerve block and reduce the incidence of complications.

## 2. Methods

### 2.1. Operating

#### 2.1.1. Single Administration of Femoral Nerve Block

First, the ultrasound image of the cross section of the femoral nerve was scanned, and the needle was inserted from the outside of the probe, which could completely show the walking process of the needle. The tip of the needle was moved to the deep surface of the femoral nerve, 5–10 ml local anesthetic was injected, and then the tip was moved to the upper surface of the femoral nerve, and then 5–10 ml local anesthetic was injected so that the femoral nerve was surrounded by the local anesthetic.

#### 2.1.2. Continuous Administration of Femoral Nerve Block

The image of the long axis of the femoral nerve was obtained by ultrasonic scanning. A 16 G trocar was used to insert the needle into the upper side of the ultrasonic probe, and the tip was placed above the lateral side of the femoral nerve. The catheter was placed from the trocar, and the front end of the catheter was driven out about 2 cm from the trocar. The trocar was pulled out, and the catheter was fixed.

### 2.2. Ultrasonic Images

In the supine position, the lower limbs were straight, and the inguinal ligament was marked between the anterior superior iliac spine and pubic tubercle. The femoral nerve is shallow, and its surface projection is about the midpoint of the inguinal ligament. Select 6–13 MHz high-frequency ultrasonic probe, place the probe parallel to the midpoint of the marking line, and adjust the ultrasonic scanning depth and gain to obtain the best image. Femoral artery can be identified firstly in ultrasound images. If multiple arteries appear on the image, translate the ultrasonic probe to the head end, and move it to the front end of the femoral artery. The femoral vein is located behind the femoral artery. If the pressure between the probe and the skin is too high, the femoral vein may be flattened and difficult to display on the ultrasound image. Usually, a triangular hyperechoic region can be seen outside the femoral artery, which is called the femoral nerve [11].

At this time, the acquired cross-sectional ultrasound image of the femoral nerve can also be placed along the running direction of the femoral artery, and then the ultrasound probe is moved to the outside until a bunch of regular strip-shaped highlight echoes appear in the image, which is the long-axis image of the femoral nerve, and the moving distance is 1–1.5 cm.

### 2.3. The Application of Upper Limb Surgery

The methods of continuous peripheral nerve block for postoperative analgesia of upper limb surgery mainly include continuous axillary brachial plexus block, which is suitable for surgery at the elbow and below the elbow and is often used for postoperative analgesia of children; continuous subclavian brachial plexus nerve block is suitable for operations above the elbow. Continuous interscalene brachial plexus block is suitable for postoperative analgesia of shoulder joint surgery and is also widely used in replantation of severed fingers of upper limbs and neurovascular anastomosis [12].

### 2.4. The Application of Lower Limb Surgery

The methods of continuous peripheral nerve block for postoperative analgesia of lower limb surgery mainly include continuous femoral nerve block and continuous lumbar nerve block. The latter has better blocking effect on the obturator nerve than the femoral nerve and is suitable for hip, thigh, and knee surgery. Continuous sciatic nerve block is mainly suitable for foot and ankle surgery.

Patients undergoing knee surgery have severe postoperative pain and long duration. Inappropriate analgesia is not conducive to functional recovery of the affected limb, and the hospitalization time is prolonged. PCIA or PCEA is usually used for postoperative analgesia, but it is not accepted by patients and medical staff because of its many side effects. Continuous peripheral nerve block can achieve analgesic effect equivalent to epidural analgesia, and adverse reactions are few and mild; it is a commonly used analgesic method after total knee arthroplasty [13].

## 3. Results and Analysis

### 3.1. Ultrasound and Nerve Block

Good nerve block not only provides the same effect as epidural analgesia after TKA but also has fewer complications. The key to successful femoral nerve block analgesia is to ensure sufficient diffusion of the local anesthetic around the nerve. However, the commonly used nerve block localization methods can only be based on anatomical markers and blind detection of heterosensory methods, which can neither observe the diffusion of local anesthetic drugs nor guarantee the effect and may damage nerves and peripheral blood vessels. Even the use of nerve stimulators is difficult to perfect the effect and may directly lead to nerve damage. Continuous femoral nerve block guided by ultrasound can clearly see the nerve structure and the position of the puncture needle and catheter, accurately place the catheter front end on the surface of the femoral nerve, ensure the distribution of local anesthetic solution around the femoral nerve, significantly improve the block effect, and reduce complications to happen.

### 3.2. Lumbar Plexus Block

Compared with the superficial brachial plexus and femoral nerve, the position of the lumbar plexus is deeper (usually 5–8 cm below the skin, and the position of patients with high body mass index is deeper), which is difficult to be identified by ultrasound. There are few reports of ultrasound-guided lumbar plexus block in clinics. Comparing the convenience, blocking effect, and adverse reactions of the short-axis in-plane technique and long-axis out-of-plane technique in total hip arthroplasty under the guidance of ultrasound, it is found that both methods can obtain good blocking effect of the lumbar plexus, and the image positioning time required by the long-axis out-of-plane technique is shorter. Ultrasound-guided long-axis out-of-plane technology is suitable for beginners because the images are easy to identify, but its disadvantage is that it is difficult to determine the needle tip position. However, the short-axis in-plane technique requires the operator to master the ultrasonic image features of different spine cross sections, which is suitable for doctors with certain operating experience and has better controllability [14].

### 3.3. Combined Nerve Stimulator

Ultrasound image is only a plane image, which cannot completely show the hierarchical relationship between organizational structures. When the nerve plexus and puncture needle cannot appear clearly in the ultrasound image at the same time or these structures are difficult to distinguish, it is necessary to constantly adjust the needle insertion direction. At this time, it may be difficult to locate by ultrasound alone, and the nerve stimulator is used in combination, and the two cooperate with each other. It may be more helpful to accurately locate the target nerve and ensure that local anesthetics are accurately injected around the nerve, thus producing good anesthetic effect [15].

Ultrasound-guided nerve stimulator is more accurate than nerve stimulation alone, with less dosage, better brachial plexus block, and lower possibility of neurovascular injury [16]. In a group of 3000 patients with peripheral nerve block without the catheter, there were 5 cases of adverse reactions when the nerve stimulator was used for localization. However, when combined with ultrasound-guided block, no adverse reactions occurred. See Table 1.

**Figure 1:**
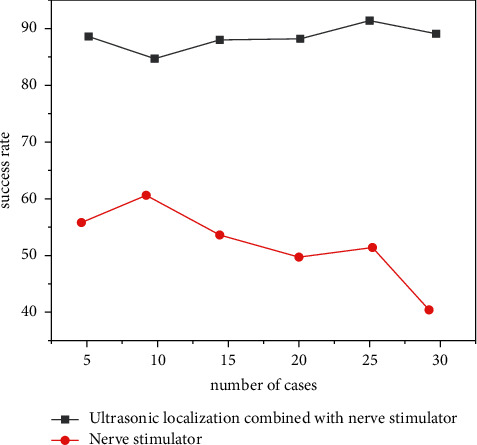
Comparison of blocking success rate between ultrasonic localization combined with the nerve stimulator and nerve stimulator.

Sixty patients undergoing upper limb surgery were identified by ultrasonic localization combined with the nerve stimulator, and retrograde subclavian and supraclavicular brachial plexus block was performed. Anesthesia took effect quickly, with fewer adverse reactions and complications [17]. For patients undergoing hand surgery during the day, continuous subclavian brachial plexus block was performed with ultrasound-guided nerve stimulator. The subclavian brachial plexus and its surrounding tissue structure, the direction of needle insertion in the plane, and the diffusion of narcotic drugs can be clearly seen. The average success rate of block is as high as 95.2%, which is obviously higher than that of nerve stimulator alone, and the success rate of recatheterization after the first failure is also improved, as shown in Figure 1. The average satisfaction rate of postoperative analgesia was 85.6%, and the average operation time was only 20 min. The subclavian artery and pleura were effectively avoided, and no complications such as pneumothorax occurred [18]. This is related to the advantages of ultrasound guidance, as shown in Table 2.

### 3.4. Pediatric Regional Block

**Figure 2:**
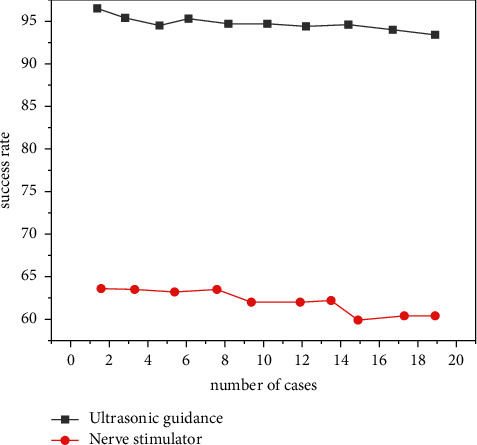
Comparison of success rate between ultrasound-guided subclavian brachial plexus block and nerve stimulator.

Regional nerve block in pediatric anesthesia is considered to be more challenging. Ultrasound-guided application in pediatric regional block is considered to be a great progress in nerve localization in pediatric regional anesthesia in recent years. Ultrasound-guided supraclavicular or subclavian brachial plexus block was used in children's upper limb surgery, and it was found that both approaches were effective. The average success rate of ultrasound-guided subclavian brachial plexus block in children aged 1-2 years was 97%, which was much higher than the average success rate of nerve stimulator localization (63%). It shows that ultrasound guidance can also improve the success rate of nerve block in children and avoid phrenic nerve and recurrent laryngeal nerve block caused by the nerve stimulator [19]. Specific data are shown in Figure 2.

At present, the ultrasound used in clinics belongs to the plane ultrasound, that is, two-dimensional ultrasound. The advantages of ultrasound-guided nerve block are that it can directly display nerves, monitor and guide the direction and depth of the puncture needle and catheter and the diffusion process of drugs in real time, and understand the data of important anatomical structures around it, which is helpful for clinical anesthesiologists to deepen their understanding of the nerve tissue structure and change the blind operation into visual operation. Anesthesia has the advantages of quick onset, long maintenance time, reduced dosage of local anesthetics, high success rate, less related complications, comfortable feeling for patients, suitability for infants, and convenience for clinical anesthesia and pain treatment.

## 4. Conclusion

Continuous peripheral nerve block is a safe and effective method for postoperative analgesia. The application of the nerve stimulator combined with ultrasonic localization technology can significantly improve the success rate of peripheral nerve block, further improve the postoperative analgesic effect, and reduce the occurrence of complications associated with catheter placement. When searching nerves and surrounding tissues, it is necessary to understand and analyze the ultrasonic characteristics of the structures of these tissues and their adjacent relationships to make an accurate judgment. Therefore, the operator is required to have rich knowledge of imaging, ultrasonic anatomy, and skilled nerve block technology. Therefore, it will be some time before ultrasound-guided regional nerve block is widely used in clinics.

## Figures and Tables

**Table 1:** Ultrasound-guided contrast nerve stimulator's response to patients with peripheral nerve block without the catheter.

Project	Number of cases	Adverse effect
Nerve stimulator	1500	5
Ultrasound-guided block	1500	0

**Table 2:** Average satisfaction and operation time of postoperative analgesia.

Project	Degree of satisfaction	Not satisfied	Operating time
Ultrasonic positioning-combined nerve stimulator	85.6	14.4	20
Nerve stimulator	52.4	47.6	40

## Data Availability

The data used to support the findings of this study are available from the corresponding author upon request.
